# Hydrogen bonds and van der Waals forces as tools for the construction of a herringbone pattern in the crystal structure of hexane-1,6-diaminium hexane-1,6-diyl bis­(hydrogen phospho­nate)

**DOI:** 10.1107/S2056989016019873

**Published:** 2017-01-01

**Authors:** Guido J. Reiss, Martin van Megen, Walter Frank

**Affiliations:** a Institut für Anorganische Chemie und Strukturchemie, Lehrstuhl II: Material- und Strukturforschung, Heinrich-Heine-Universität Düsseldorf, Universitätsstrasse 1, D-40225 Düsseldorf, Germany

**Keywords:** crystal structure, hydrogen bonding, phospho­nates, herringbone pattern, crystal engineering

## Abstract

The asymmetric unit of the title salt, [H_3_N(CH_2_)_6_NH_3_][(HO)O_2_P(CH_2_)_6_PO_2_(OH)], consists of one half of a hexane-1,6-diaminium dication and one half of a hexane-1,6-diyl bis­(hydrogen phospho­nate) dianion. Both are located around different centres of inversion (Wyckoff sites: 2*a* and 2*d*) of the space group *P*2_1_/*c*. The shape of the hexane-1,6-diaminium cation is best described as a double hook. Both aminium groups as well as the two attached CH_2_ groups are turned out from the plane of the central four C atoms. In contrast, all six C atoms of the dianion are almost in a plane. The hydrogen phospho­nate (–PO_3_H) groups of the anions and the aminium groups of the cations form two-dimensional O—H⋯ and O—H⋯N hydrogen-bonded networks parallel to the *ac* plane, built up from ten-membered and twelve-membered ring motifs with graph-set descriptors *R*_3_^3^(10) and *R*_5_^4^(12), respectively. These networks are linked by the alkyl­ene chains of the anions and cations. The resulting three-dimensional network shows a herringbone pattern, which resembles the parent structures 1,6-di­amino­hexane and hexane-1,6-di­phospho­nic acid.

## Abstract 2

The solid-state structure of the title salt, [H_3_N(CH_2_)_6_NH_3_][(HO)O_2_P(CH_2_)_6_PO_2_(OH)], possesses a herringbone motif as a consequence of the inter­play of strong hydrogen bonds and non-covalent inter­actions.

## Chemical context   

Salts which comprise organo­phospho­nate anions and organic cations, *e.g.* protonated primary (Mahmoudkhani & Langer, 2002*a* ▸,*b* ▸,*c* ▸), secondary (Wheatley *et al.*, 2001 ▸) or tertiary amines (Kan & Ma, 2011 ▸) are of growing inter­est in supra­molecular chemistry and crystal engineering. Compounds of this type possess inter­esting topologies and an extended structural diversity. Furthermore, they seem to be feasible model systems for metal phospho­nates as they exhibit similar structural characteristics. Most of these salt-type solids show extended hydrogen-bonded networks which are characterized by a rich diversity of strong charge-supported hydrogen bonds (Aakeröy & Seddon, 1993 ▸; Białek *et al.*, 2013 ▸) besides some weaker inter­molecular inter­actions (van Megen *et al.*, 2016**a* ▸*,*b* ▸).

A search in the Cambridge Structure Database (Groom *et al.*, 2016 ▸) yielded more than 180 entries for the hexane-1,6-diaminium dication (*H16AH*). At this point it is not our aim to review all these structures, but we think it is worth highlighting some important classes of compounds and applications. The structures and properties of many simple salts of *H16AH*, like halides (van Blerk & Kruger, 2008 ▸), acetates (Paul & Kubicki, 2009 ▸) and salts with more complex inorganic anions such as hexa­fluorido­silcate (Ouasri *et al.*, 2014 ▸), tetra­iodide (Reiss & van Megen, 2012 ▸) or di­hydrogen arsenate (Wilkinson & Harrison, 2007 ▸) have been extensively studied. Moreover, the *H16AH* dication is well known for its use in crystal engin­eering of hydrogen-bonded solids which contain unstable species (Frank & Reiss, 1997 ▸), in supra­molecular chemistry (Assaf & Nau, 2015 ▸), as a tecton for the construction of layered materials (Bujoli-Doeuff *et al.*, 2012 ▸), or as a cationic template for novel complex systems (Holtby *et al.*, 2007 ▸). Finally, it should be stressed out that the *H16AH* cation is applied in the context of nylon-based hybride materials (Boncel *et al.*, 2014 ▸).
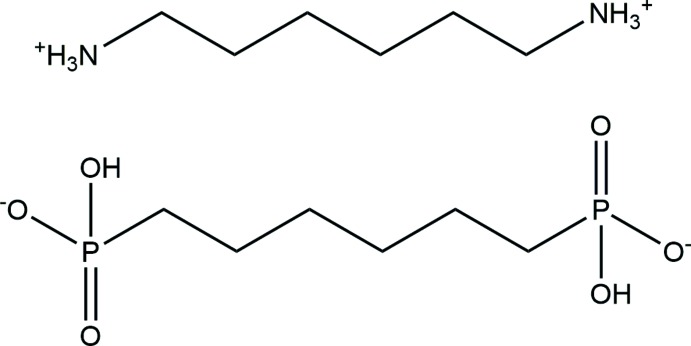


This contribution is part of an ongoing study regarding the structural chemistry of alkane-*α*,*ω*-di­phospho­nic acids (van Megen *et al.*, 2015 ▸) and their organic aminium salts (van Megen *et al.*, 2016*a* ▸,*b* ▸).

## Structural commentary   

The asymmetric unit of [H_3_N(CH_2_)_6_NH_3_][(HO)O_2_P(CH_2_)_6_PO_2_(OH)] consists of one half of an *H16AH* dication and one half of a hexane-1,6-diyl bis(hydrogen phospho­nate) dianion (*16PHOS*). Both ions are located around different inversion centres of space group type *P*2_1_/*c* (Wyckoff sites 2*a* and 2*d*, respectively). Bond lengths and angles in the dication as well as in the dianion are in the expected ranges (Table 1 ▸).

**Figure 1:**
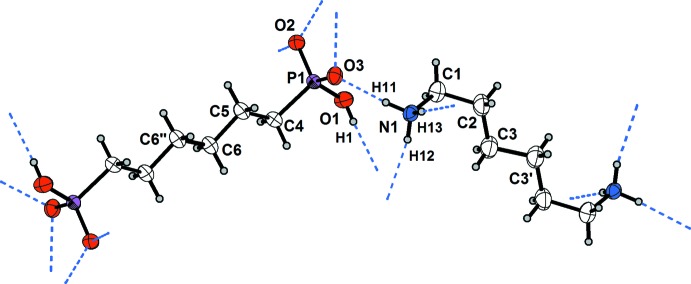
The *H16AH* cation and the *16PHOS* anion are shown together with their hydrogen bonds. Displacement ellipsoids are drawn at the 50% probability level; hydrogen atoms are drawn as spheres with arbitrary radii. [Symmetry codes: (′) −*x*, −*y*, −*z*; (′′) 1 − *x*, 1 − *y*, 1 − *z*.]

As shown in Fig. 1 ▸, the cation has a conformation best described as a double hook. In detail, atom C1 is turned out from the plane of the central four carbon atoms by about 6° (Table 1 ▸), whereas atom N1 is turned out significantly from the plane defined by the central four carbon atoms [N1—C1—C2—C3 = 69.9 (3)°]. The individual conformation of the cationic diaminium tecton seems to be a compromise between an effort to form the most stable conformation on the one hand, and inter­molecular inter­actions, namely hydrogen bonding and van der Waals inter­actions, on the other hand (Frank & Reiss, 1996 ▸, 1997 ▸).

The conformation of the anion is that of the energetically most stable all-*transoid* conformation of the hexane-1,6-diyl moiety (r.m.s. of the six carbon atoms and two phospho­rus atoms: 0.2643 Å), also expressed by the almost perfect *anti*-periplanar arrangement of each CH_2_ group (*cf*. the torsion angles in Table 1 ▸). A detailed view of the hydrogen phospho­nate groups shows the P—OH distance of 1.5817 (14) Å to be greater than the two other P—O distances [1.4977 (13) and 1.5112 (13) Å].

## Supra­molecular features   

Within the crystal of the title compound, the aminium groups of the cations as well as the hydrogen phospho­nate groups of the anions form hydrogen bonds with adjacent ions. In detail, each hydrogen atom of the NH_3_ group and the OH group of the hydrogen phospho­nate moiety donates a single hydrogen bond to a phosphoryl oxygen atom (Fig. 1 ▸), whereby each phosphoryl oxygen atom accepts two hydrogen bonds.

**Figure 2:**
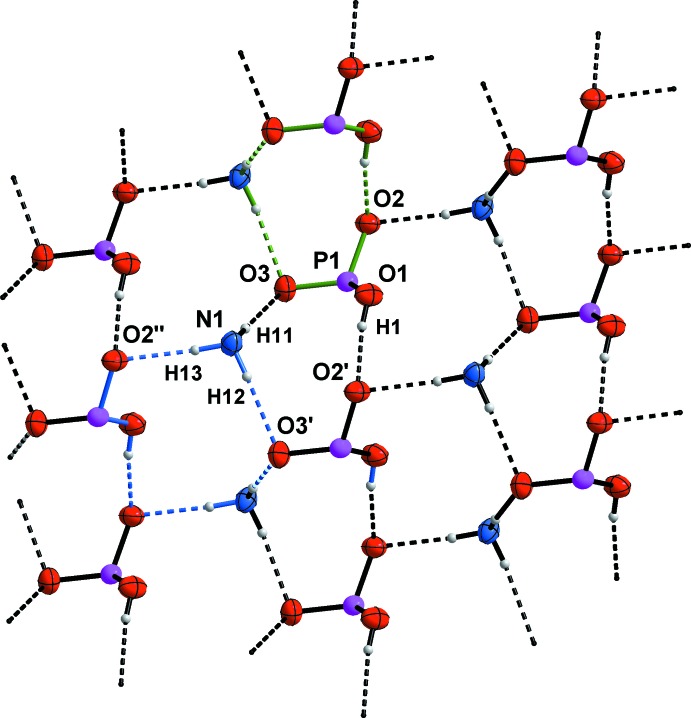
The two-dimensional hydrogen-bonded network composed of aminium and hydrogen phospho­nate groups parallel to the *ac* plane. The 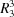
(10) graph-set motif is indicated by green bonds and the 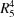
(12) motif with blue bonds. [Symmetry codes: (′) *x*, −*y* + 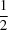, *z* − 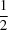; (′′) *x* − 1, −*y* + 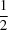, *z* − 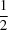.]

Anions and cations are connected by medium strong to strong, charge-supported N—H⋯O and O—H⋯O hydrogen bonds (Steiner, 2002 ▸; Table 2 ▸). The hydrogen-bonding inter­actions help to construct a two-dimensional network which propagates parallel to the *ac* plane (Fig. 2 ▸). This network contains two characteristic types of meshes (Fig. 2 ▸), which can be classified as ten-membered and twelve-membered hydrogen-bonded ring motifs with the first level graph-set descriptors 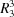
(10) and 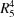
(12), respectively (Etter *et al.*, 1990 ▸). It is remarkable that the structure of NH_4_C_10_H_21_PO_2_OH (Boczula *et al.*, 2012 ▸) possesses layers with a very similar topology [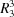
(10) and 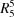
(12)].

Along the *b* axis of the unit cell, these hydrogen-bonded networks are linked by the alkyl­ene chains of the anions as well as the cations, forming a three-dimensional network with a typical herringbone pattern.

We have already shown that α,ω-diaminiumalkane tectons support the formation of salts with tailored, linear polyiodides (Reiss & Engel, 2002 ▸) showing a herringbone pattern with alternating cations and anions. Thus, the title structure is a further example for both the robustness of the herringbone motif and the structure-directing properties of α,ω-functionalized alkylene tectons.

**Figure 3:**
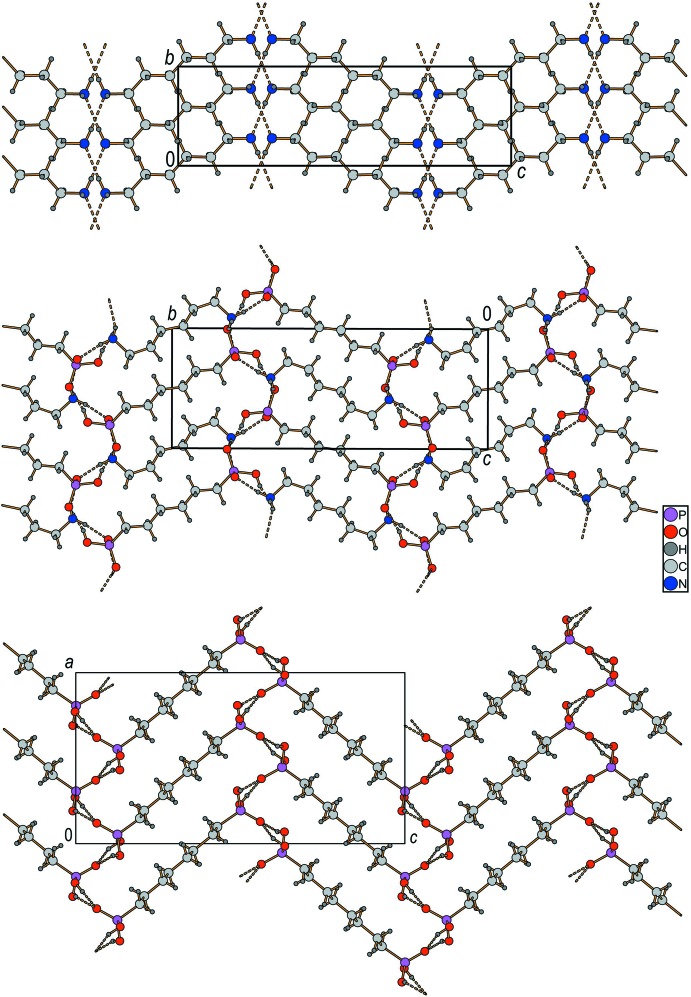
Comparison of the herringbone pattern of 1,6-di­amino­hexane (upper part), 1,6-hexane-di­phospho­nic acid (lower part), and the title compound (middle part).

A comparison with the ‘parent’ structures, namely those of 1,6-di­amino­hexane (Thalladi *et al.*, 2000 ▸) and hexane-1,6-di­phospho­nic acid (van Megen *et al.*, 2015 ▸) seems useful. A characteristic feature of each herringbone motif is the angle of the fishbones to each other. It is not surprising, then, that this angle in the title crystal structure is almost the average of those found for the parent structures (Fig. 3 ▸), which is another proof of the usefulness of α,ω-diaminiumalkane tectons in crystal engineering.

## Related structures   

For related hydrogen phospho­nates, phospho­nates and bis(phospho­nates), see: Boczula *et al.* (2012 ▸); Ferguson *et al.* (1998 ▸); Fu *et al.* (2004 ▸); Fuller & Heimer (1995 ▸); Glidewell *et al.* (2000 ▸); Kan & Ma (2011 ▸); Mahmoudkhani & Langer (2002*a* ▸,*b* ▸,*c* ▸); Plabst *et al.* (2009 ▸); van Megen *et al.* (2016*a* ▸,*b* ▸); Wheatley *et al.* (2001 ▸).

For related hexane-1,6-diaminium salts, see: Assaf & Nau (2015 ▸); Boncel *et al.* (2014 ▸); Bujoli-Doeuff *et al.* (2012 ▸); Blerk & Kruger (2008 ▸); Frank & Reiss (1997 ▸); Holtby *et al.* (2007 ▸); Wilkinson & Harrison (2007 ▸); van Megen *et al.* (2015 ▸).

For closely related hydrogen-bonded compounds with a herringbone pattern, see: Thalladi *et al.* (2000 ▸); van Megen *et al.* (2016*a* ▸).

## Synthesis and crystallization   

For the preparation of the title compound, equimolar qu­an­ti­ties (0.5 mmol) of hexane-1,6-di­amine (58.1 mg) and hexane-1,6-bis­phospho­nic acid (123.1 mg) were dissolved in methanol, separately. The solutions were mixed and the resulting white precipitate was then dissolved in distilled water. Within several days, colourless crystals were obtained in an open petri dish by slow evaporation of the solvent. Hexane-1,6-di­amine was purchased from commercial sources and hexane-1,6-bis­phospho­nic acid was synthesized according to the literature (Schwarzenbach & Zurc, 1950 ▸; Moedritzer & Irani, 1961 ▸; Griffith *et al.*, 1998 ▸).

Elemental analysis: C_12_H_32_N_2_O_6_P_2_ (362.33): calculated C 39.8, H 8.9, N 7.7; found C 39.8, H 9.7, N 8.4., m.p.: 501 K.

## IR and Raman spectra   

**Figure 4:**
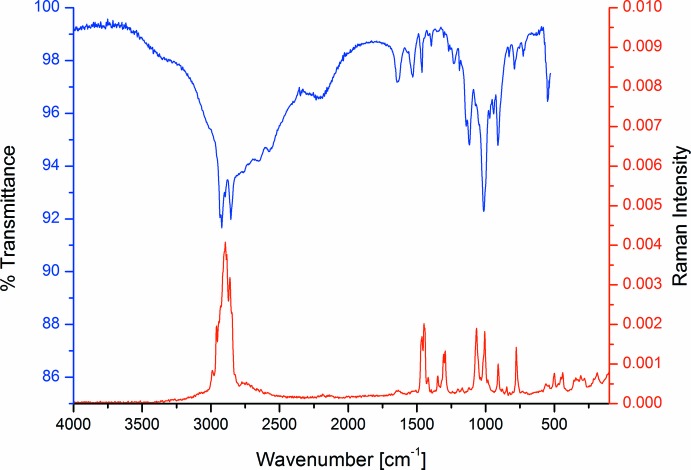
The IR (blue) and Raman (red) spectra of the title compound.

The IR and Raman spectra of the title compound are shown in Fig. 4 ▸. The vibration spectra of the title compound are in excellent accord with those of NH_4_C_10_H_21_PO_2_OH (Boczula *et al.*, 2012 ▸). This is not particularly surprising as both structures are closely related, including the hydrogen-bonding schemes. Since Boczula *et al.* presented a detailed discussion of the spectra, we do not include a repeated discussion. An additional, often neglected feature of such IR spectra are the broad bands associated with the O—H stretching vibration indicating strong hydrogen bonds (Hadži, 1965 ▸; Baran *et al.*, 1989 ▸). A detailed discussion has also been reported very recently (van Megen *et al.*, 2016*a* ▸) for this feature. In the IR spectrum of the title compound, the maxima of the so called A, B and C bands can be estimated to be at 2750, 2200 and 1600 cm^−1^.

## Refinement   

Crystal data, data collection and structure refinement details are summarized in Table 3 ▸. All hydrogen atoms bound to either nitro­gen or oxygen atoms were identified in difference syntheses and refined without any geometric constraints or restraints with individual *U*_iso_(H) values. Carbon-bound hydrogen atoms were included using a riding model (AFIX 23 option of the *SHELX* program for the methyl­ene groups and AFIX 43 option for the methine groups).

## Supplementary Material

Crystal structure: contains datablock(s) I, publication_text. DOI: 10.1107/S2056989016019873/wm5345sup1.cif

Structure factors: contains datablock(s) I. DOI: 10.1107/S2056989016019873/wm5345Isup2.hkl

CCDC reference: 1522538

Additional supporting information: crystallographic information; 3D view; checkCIF report

## Figures and Tables

**Table 1:** Selected geometric parameters (Å, °)

P1—O3	1.4977 (13)	P1—O1	1.5817 (14)
P1—O2	1.5112 (13)		
			
O3—P1—O2	114.23 (8)	O3—P1—C4	111.37 (9)
O3—P1—O1	111.27 (8)	O2—P1—C4	109.71 (8)
O2—P1—O1	105.83 (8)	O1—P1—C4	103.79 (8)
			
N1—C1—C2—C3	69.9 (3)	P1—C4—C5—C6	−177.99 (15)
C1—C2—C3—C3^i^	174.2 (3)	C4—C5—C6—C6^ii^	178.7 (2)

Symmetry codes: (i) 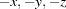; (ii) 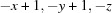.

**Table 2:** Hydrogen-bond geometry (Å, °)

*D*—H⋯*A*	*D*—H	H⋯*A*	*D*⋯*A*	*D*—H⋯*A*
N1—H11⋯O3	0.89 (2)	1.90 (2)	2.782 (2)	168 (2)
N1—H12⋯O3^′^	0.87 (3)	2.05 (3)	2.905 (2)	165 (2)
N1—H13⋯O2^′′^	0.90 (2)	1.94 (2)	2.828 (2)	170 (2)
O1—H1⋯O2^′^	0.81 (3)	1.76 (3)	2.5546 (19)	168 (3)

Symmetry codes: (′) 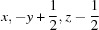; (′′) 
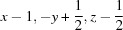
.

**Table 3:** Experimental details

Crystal data	
Chemical formula	C_6_H_18_N_2_^2+^·C_6_H_14_O_6_P_2_^2−^
*M*_r_	362.33
Crystal system, space group	Monoclinic, *P*2_1_/*c*
Temperature (K)	292
*a*, *b*, *c* (Å)	5.88242 (16), 20.2162 (5), 7.7574 (2)
β (°)	98.090 (3)
*V* (Å^3^)	913.33 (4)
*Z*	2
Radiation type	Mo *K*α
μ (mm^−1^)	0.27
Crystal size (mm)	0.40 × 0.20 × 0.12

Data collection	
Diffractometer	Oxford Diffraction Xcalibur with Eos detector
Absorption correction	Multi-scan (*CrysAlis PRO*; Oxford Diffraction, 2006 ▸)
*T*_min_, *T*_max_	0.898, 1.000
No. of measured, independent and observed [*I* > 2σ(*I*)] reflections	14194, 2779, 2339
*R*_int_	0.022
(sin θ/λ)_max_ (Å^−1^)	0.714

Refinement	
*R*[*F* ^2^ > 2σ(*F* ^2^)], *wR*(*F* ^2^), *S*	0.047, 0.098, 1.02
No. of reflections	2779
No. of parameters	116
H-atom treatment	H atoms treated by a mixture of independent and constrained refinement
Δρ_max_, Δρ_min_ (e Å^−3^)	0.64, −0.28

Computer programs: *CrysAlis PRO* (Oxford Diffraction, 2006 ▸), *SHELXT* (Sheldrick, 2015*a* ▸), *SHELXL2014* (Sheldrick, 2015*b* ▸), *DIAMOND* (Brandenburg, 2015 ▸) and *publCIF* (Westrip, 2010 ▸).

## supplementary crystallographic information

### Crystal data

**Table:**

C_6_H_18_N_2_^2+^·C_6_H_14_O_6_P_2_^2^^−^	*D*_x_ = 1.318 Mg m^−^^3^
*M**_r_* = 362.33	Melting point: 501 K
Monoclinic, *P*2_1_/*c*	Mo *K*α radiation, λ = 0.71073 Å
*a* = 5.88242 (16) Å	Cell parameters from 8526 reflections
*b* = 20.2162 (5) Å	θ = 3.0–33.9°
*c* = 7.7574 (2) Å	µ = 0.27 mm^−^^1^
β = 98.090 (3)°	*T* = 292 K
*V* = 913.33 (4) Å^3^	Block, colorless
*Z* = 2	0.40 × 0.20 × 0.12 mm
*F*(000) = 392	

### Data collection

**Table:**

Oxford Diffraction Xcalibur with Eos detector diffractometer	2779 independent reflections
Radiation source: (Mo) X-ray Source	2339 reflections with *I* > 2σ(*I*)
Detector resolution: 16.2711 pixels mm^-1^	*R*_int_ = 0.022
ω scans	θ_max_ = 30.5°, θ_min_ = 3.3°
Absorption correction: multi-scan (*CrysAlis PRO*; Oxford Diffraction, 2006)	*h* = −8→8
*T*_min_ = 0.898, *T*_max_ = 1.000	*k* = −28→28
14194 measured reflections	*l* = −11→10

### Refinement

**Table:**

Refinement on *F*^2^	0 restraints
Least-squares matrix: full	Hydrogen site location: mixed
*R*[*F*^2^ > 2σ(*F*^2^)] = 0.047	H atoms treated by a mixture of independent and constrained refinement
*wR*(*F*^2^) = 0.098	*w* = 1/[σ^2^(*F*_o_^2^) + (0.018*P*)^2^ + 1.*P*] where *P* = (*F*_o_^2^ + 2*F*_c_^2^)/3
*S* = 1.02	(Δ/σ)_max_ < 0.001
2779 reflections	Δρ_max_ = 0.64 e Å^−^^3^
116 parameters	Δρ_min_ = −0.28 e Å^−^^3^

### Special details

**Table:**

Geometry. All esds (except the esd in the dihedral angle between two l.s. planes) are estimated using the full covariance matrix. The cell esds are taken into account individually in the estimation of esds in distances, angles and torsion angles; correlations between esds in cell parameters are only used when they are defined by crystal symmetry. An approximate (isotropic) treatment of cell esds is used for estimating esds involving l.s. planes.

### Fractional atomic coordinates and isotropic or equivalent isotropic displacement parameters (Å^2^)

**Table:**

	*x*	*y*	*z*	*U*_iso_*/*U*_eq_	
N1	−0.0383 (3)	0.18730 (8)	0.0899 (2)	0.0330 (3)	
H11	0.005 (4)	0.2266 (12)	0.135 (3)	0.043 (6)*	
H12	0.024 (4)	0.1833 (11)	−0.006 (3)	0.047 (7)*	
H13	−0.190 (4)	0.1883 (11)	0.054 (3)	0.043 (6)*	
C1	0.0201 (4)	0.13413 (10)	0.2199 (3)	0.0446 (5)	
H1A	−0.0407	0.1459	0.3257	0.053*	
H1B	0.1858	0.1315	0.2478	0.053*	
C2	−0.0708 (4)	0.06670 (10)	0.1609 (3)	0.0501 (5)	
H2A	−0.0529	0.0373	0.2607	0.060*	
H2B	−0.2338	0.0706	0.1201	0.060*	
C3	0.0423 (4)	0.03569 (11)	0.0201 (4)	0.0532 (6)	
H3A	0.2072	0.0352	0.0552	0.064*	
H3B	0.0107	0.0622	−0.0848	0.064*	
P1	0.40040 (8)	0.30335 (2)	0.30797 (6)	0.02754 (11)	
O1	0.5147 (2)	0.23351 (7)	0.28712 (19)	0.0388 (3)	
H1	0.490 (4)	0.2187 (12)	0.190 (3)	0.051 (7)*	
O2	0.4820 (2)	0.32500 (6)	0.49279 (16)	0.0357 (3)	
O3	0.1446 (2)	0.29998 (6)	0.26262 (18)	0.0359 (3)	
C4	0.5251 (3)	0.35522 (9)	0.1593 (2)	0.0367 (4)	
H4A	0.6898	0.3568	0.1952	0.044*	
H4B	0.4988	0.3351	0.0447	0.044*	
C5	0.4345 (4)	0.42525 (9)	0.1453 (3)	0.0411 (4)	
H5A	0.4665	0.4467	0.2580	0.049*	
H5B	0.2693	0.4243	0.1120	0.049*	
C6	0.5441 (4)	0.46516 (10)	0.0111 (3)	0.0457 (5)	
H6A	0.7089	0.4664	0.0462	0.055*	
H6B	0.5155	0.4427	−0.1004	0.055*	

### Atomic displacement parameters (Å^2^)

**Table:**

	*U*^11^	*U*^22^	*U*^33^	*U*^12^	*U*^13^	*U*^23^
N1	0.0291 (7)	0.0326 (8)	0.0383 (9)	−0.0038 (6)	0.0077 (6)	−0.0065 (6)
C1	0.0472 (11)	0.0334 (10)	0.0519 (12)	−0.0008 (8)	0.0029 (9)	−0.0008 (9)
C2	0.0533 (12)	0.0333 (10)	0.0656 (15)	−0.0034 (9)	0.0151 (11)	0.0004 (10)
C3	0.0521 (13)	0.0383 (11)	0.0710 (16)	−0.0045 (10)	0.0152 (12)	−0.0072 (11)
P1	0.0292 (2)	0.0280 (2)	0.0258 (2)	−0.00367 (16)	0.00522 (15)	0.00149 (16)
O1	0.0460 (8)	0.0380 (7)	0.0322 (7)	0.0093 (6)	0.0050 (6)	0.0007 (6)
O2	0.0410 (7)	0.0384 (7)	0.0277 (6)	−0.0069 (5)	0.0052 (5)	−0.0010 (5)
O3	0.0297 (6)	0.0351 (7)	0.0425 (7)	−0.0028 (5)	0.0042 (5)	−0.0021 (6)
C4	0.0428 (10)	0.0372 (9)	0.0321 (9)	−0.0055 (8)	0.0119 (8)	0.0024 (7)
C5	0.0538 (12)	0.0339 (9)	0.0381 (10)	−0.0053 (8)	0.0151 (9)	0.0054 (8)
C6	0.0619 (13)	0.0365 (10)	0.0412 (11)	−0.0099 (9)	0.0165 (10)	0.0066 (8)

### Geometric parameters (Å, º)

**Table:**

N1—C1	1.481 (3)	P1—O2	1.5112 (13)
N1—H11	0.89 (2)	P1—O1	1.5817 (14)
N1—H12	0.87 (3)	P1—C4	1.7907 (18)
N1—H13	0.90 (2)	O1—H1	0.81 (3)
C1—C2	1.511 (3)	C4—C5	1.511 (3)
C1—H1A	0.9700	C4—H4A	0.9700
C1—H1B	0.9700	C4—H4B	0.9700
C2—C3	1.494 (3)	C5—C6	1.529 (3)
C2—H2A	0.9700	C5—H5A	0.9700
C2—H2B	0.9700	C5—H5B	0.9700
C3—C3^i^	1.544 (4)	C6—C6^ii^	1.503 (4)
C3—H3A	0.9700	C6—H6A	0.9700
C3—H3B	0.9700	C6—H6B	0.9700
P1—O3	1.4977 (13)		
			
C1—N1—H11	110.7 (14)	O3—P1—O1	111.27 (8)
C1—N1—H12	115.2 (15)	O2—P1—O1	105.83 (8)
H11—N1—H12	107 (2)	O3—P1—C4	111.37 (9)
C1—N1—H13	110.5 (14)	O2—P1—C4	109.71 (8)
H11—N1—H13	108.3 (19)	O1—P1—C4	103.79 (8)
H12—N1—H13	105 (2)	P1—O1—H1	113.8 (18)
N1—C1—C2	114.23 (18)	C5—C4—P1	115.00 (13)
N1—C1—H1A	108.7	C5—C4—H4A	108.5
C2—C1—H1A	108.7	P1—C4—H4A	108.5
N1—C1—H1B	108.7	C5—C4—H4B	108.5
C2—C1—H1B	108.7	P1—C4—H4B	108.5
H1A—C1—H1B	107.6	H4A—C4—H4B	107.5
C3—C2—C1	115.14 (19)	C4—C5—C6	111.41 (17)
C3—C2—H2A	108.5	C4—C5—H5A	109.3
C1—C2—H2A	108.5	C6—C5—H5A	109.3
C3—C2—H2B	108.5	C4—C5—H5B	109.3
C1—C2—H2B	108.5	C6—C5—H5B	109.3
H2A—C2—H2B	107.5	H5A—C5—H5B	108.0
C2—C3—C3^i^	112.1 (2)	C6^ii^—C6—C5	113.6 (2)
C2—C3—H3A	109.2	C6^ii^—C6—H6A	108.8
C3^i^—C3—H3A	109.2	C5—C6—H6A	108.8
C2—C3—H3B	109.2	C6^ii^—C6—H6B	108.8
C3^i^—C3—H3B	109.2	C5—C6—H6B	108.8
H3A—C3—H3B	107.9	H6A—C6—H6B	107.7
O3—P1—O2	114.23 (8)		
			
N1—C1—C2—C3	69.9 (3)	O1—P1—C4—C5	176.71 (15)
C1—C2—C3—C3^i^	174.2 (3)	P1—C4—C5—C6	−177.99 (15)
O3—P1—C4—C5	56.90 (17)	C4—C5—C6—C6^ii^	178.7 (2)
O2—P1—C4—C5	−70.57 (17)		

Symmetry codes: (i) −*x*, −*y*, −*z*; (ii) −*x*+1, −*y*+1, −*z*.

### Hydrogen-bond geometry (Å, º)

**Table:**

*D*—H···*A*	*D*—H	H···*A*	*D*···*A*	*D*—H···*A*
N1—H11···O3	0.89 (2)	1.90 (2)	2.782 (2)	168 (2)
N1—H12···O3^iii^	0.87 (3)	2.05 (3)	2.905 (2)	165 (2)
N1—H13···O2^iv^	0.90 (2)	1.94 (2)	2.828 (2)	170 (2)
O1—H1···O2^iii^	0.81 (3)	1.76 (3)	2.5546 (19)	168 (3)

Symmetry codes: (iii) *x*, −*y*+1/2, *z*−1/2; (iv) *x*−1, −*y*+1/2, *z*−1/2.
